# Machine-learning prediction of BMI change among doctors and nurses in North China during the COVID-19 pandemic

**DOI:** 10.3389/fnut.2023.1019827

**Published:** 2023-01-26

**Authors:** Qihe Wang, Haiyun Chu, Pengfeng Qu, Haiqin Fang, Dong Liang, Sana Liu, Jinliang Li, Aidong Liu

**Affiliations:** ^1^Department of Nutrition Division I, China National Center for Food Safety Risk Assessment, Beijing, China; ^2^Public Health Institute of Harbin Medical University, Harbin, China; ^3^Department of General Internal Medicine, Harbin Sixth Hospital, Harbin, China

**Keywords:** COVID-19 pandemic, BMI change, doctors and nurses, machine learning, China

## Abstract

**Objective:**

The COVID-19 pandemic has become a major public health concern over the past 3 years, leading to adverse effects on front-line healthcare workers. This study aimed to develop a Body Mass Index (BMI) change prediction model among doctors and nurses in North China during the COVID-19 pandemic, and further identified the predicting effects of lifestyles, sleep quality, work-related conditions, and personality traits on BMI change.

**Methods:**

The present study was a cross-sectional study conducted in North China, during May-August 2022. A total of 5,400 doctors and nurses were randomly recruited from 39 COVID-19 designated hospitals and 5,271 participants provided valid responses. Participants’ data related to social-demographics, dietary behavior, lifestyle, sleep, personality, and work-related conflicts were collected with questionnaires. Deep Neural Network (DNN) was applied to develop a BMI change prediction model among doctors and nurses during the COVID-19 pandemic.

**Results:**

Of participants, only 2,216 (42.0%) individuals kept a stable BMI. Results showed that personality traits, dietary behaviors, lifestyles, sleep quality, burnout, and work-related conditions had effects on the BMI change among doctors and nurses. The prediction model for BMI change was developed with a 33-26-20-1 network framework. The DNN model achieved high prediction efficacy, and values of *R*^2^, MAE, MSE, and RMSE for the model were 0.940, 0.027, 0.002, and 0.038, respectively. Among doctors and nurses, the top five predictors in the BMI change prediction model were unbalanced nutritional diet, poor sleep quality, work-family conflict, lack of exercise, and soft drinks consumption.

**Conclusion:**

During the COVID-19 pandemic, BMI change was highly prevalent among doctors and nurses in North China. Machine learning models can provide an automated identification mechanism for the prediction of BMI change. Personality traits, dietary behaviors, lifestyles, sleep quality, burnout, and work-related conditions have contributed to the BMI change prediction. Integrated treatment measures should be taken in the management of weight and BMI by policymakers, hospital administrators, and healthcare workers.

## 1. Introduction

Over the past 3 years, the coronavirus disease 2019 (COVID-19) pandemic has become a major public health concern (1). In China, the government has promulgated and implemented a series of dramatic containment measures against the COVID-19 pandemic and protect people’s life safety and health. The COVID-19 designated hospitals and Fangcang shelter hospitals for centralized treatment (2), strict locked-down policies (3, 4), frequent nucleic acid detection (5), and effective COVID-19 vaccines (6–9) are now becoming commonplace. The COVID-19 pandemic, an onset of a sudden and immediately life-threatening infectious disease, has led to extraordinary amounts of pressure on the front-line healthcare workers, particularly doctors and nurses. A myriad of stressors, such as heavy workload, unstable working setting, strict safety measures, inadequate personal equipment, physical exhaustion, infection risk, and distress about the loss of patient lives, may have dramatic effects on their physical and mental health (10, 11). Weight and Body Mass Index (BMI) changes are the most directly impacted physical symptoms (12). Epidemiological and clinical studies indicated that both substantial weight gain and weight loss would increase the lifetime risk of various diseases due to endocrine abnormality (13–16). Therefore, it is of great significance to determine the risk factors and develop a prediction model of BMI change among Chinese doctors and nurses.

BMI change is vulnerable to multiple variables, including lifestyles, sleep quality, work-related conditions, as well as personality traits. During the COVID-19 pandemic, studies revealed that millions of households had suffered food habits, lifestyles, sleep quality, and weight changes over several months (17–20). Unhealthy dietary pattern is characterized by a high-fat diet, high-salt diet, high-carbohydrate diet, poor dietary regularity, etc. which is a leading cause of poor weight management. Meanwhile, the isolation and lockdown during COVID-19 bring a variety of mental problems and emotional eating behaviors. A survey during COVID-19 showed increased emotional eating associated with psychological and social distress (21). Emotional eating has been suggested to be one major predictor for 7-year waist circumference and BMI changes, as well as the subsequent development of obesity (22). Another important risk factor for BMI change is insufficient exercise. The frequency of exercise decreased during the pandemic, especially after the lockdown. A dose-response relationship between exercise and the prevention of overweight or obesity, chronic diseases, and mental health is well-documented (23, 24). Previous studies suggested that poor sleep quality was a major risk factor for weight and BMI changes (17, 25). Sleep latency, sleep duration, insomnia, and sleep disturbances are associated with BMI change. Moreover, the COVID-19 pandemic has made healthcare workers busier than before, bringing greater work stress. Work stress and conflict have a great impact on their physical and mental health. A review of 44 studies reported that high levels of working stress during outbreaks were in 18.1–80.1%, and severe anxiety, depressive symptoms and insomnia symptoms were in 45%, 27.5–50.7%, and 34–36.1%, respectively (26). The emergency and work overload make it difficult for doctors and nurses to adapt to a new setting, balance work and family, and lead to job burnout, poor sleep, and significant body weight change (27–30). Additionally, personality psychologists point out that personality traits contribute to how people respond and behave during a pandemic. Individuals with different personality traits have different working attitude, performance, and coping styles dealing with problems (31, 32). For example, individuals with neuroticism are not good at dealing with conflicts, problems and emotions at work, leading to physical and mental exhaustion; while agreeableness played a moderating role between low social support and psychological distress (33, 34). Besides, personality traits play an important role in eating behaviors (35), alcohol use (36), physical activity (37), etc. All these factors contribute to body weight and BMI change. Given the importance of medical service, it is very important and urgent to pay attention to the effects of personality traits, lifestyle, burnout, and work-related conflict on the changes of BMI among doctors and nurses.

Numerous studies have provided evidence for the changes of dietary behaviors, lifestyle, and work-related situations during the COVID-19 pandemic (19, 38). Consequently, the increased prevalence of physical symptoms was reported (10). Understanding the impact of the COVID-19 pandemic on weight management of healthcare workers is crucial. However, few research on the BMI change of doctors and nurses over the past 3 years. And so far, existing studies explored the risk factors of weight and BMI changes based on statistical methods. Recent studies strengthen the evidence that machine learning (ML) is commonly applied in all areas of modern human life (39, 40). Artificial Neural Network (ANN) is one of the essential tools that has been extensively studied in the field of diagnosis of various diseases. An ANN draws inspiration from the biological neural networking system, aimed at processing a large amount of data simultaneously. The rationale behind this approach is that ANN activations are themselves a product of non-linear transformations. Deep Neural Network (DNN) is typically used to make a prediction through a series of layers, each of which combines an affine operation and a non-linearity. Studies showed that DNN models could provide an automated identification mechanism for various diseases, such as cardiovascular disease (41), diabetic retinopathy (42), neurological disorders (43), etc. Hence, in order to provide insights, augment prevention, and reduce risks in weight management among healthcare workers, there are great benefits of applying machine learning technology to develop a prediction model of BMI change among doctors and nurses during the COVID-19 pandemic.

The current study recruited doctors and nurses from COVID-19 designated hospitals as samples, applied Deep Neural Network (DNN) to develop a BMI change prediction model, and further identified the predicting effects of lifestyles, sleep quality, work-related conditions, and personality traits on the BMI change, which provided the scientific basis for mental and physical health improvement among doctors and nurses during the COVID-19 pandemic.

## 2. Materials and methods

### 2.1. Sample and procedures

The present study was a cross-sectional study conducted in North China, during May-August 2022. A total of 5,400 doctors and nurses were randomly recruited from 39 COVID-19 designated hospitals in North China. Firstly, 39 COVID-19 designated hospitals were selected from 8 provinces and municipalities in North China, including Heilongjiang, Liaoning, Jilin, Hebei, Shandong, Shanxi, Beijing, and Tianjin. Secondly, we calculated the distribution of healthcare workers from these hospitals as the proportion of participants. Thirdly, based on the proportion of participants, we randomly recruited participants from these designated hospitals in North China. Fourthly, each hospital temporarily set up a special interview office in the administration building for this survey, and two well-trained investigators introduced the purpose, content, principles, etc. of this study to the participants. The face-to-face interview happened during breaks or after hours, depending on the participants’ inclinations. The data were saved immediately after the survey. Finally, 5,400 participants were recruited to participate in this survey. Samples eligible for inclusion in this study were age ≥ 18 years old and voluntary participation. Exclusion criteria included voluntary problems, diseases (e.g., coronary disease, diabetes, hyperthyroidism…), medicine taking (e.g., hypoglycemic and hypolipidemic agents…), pregnant women, and other special physiological conditions (e.g., menopausal women, individuals in strict weight loss period…). In this investigation, researchers clearly explained the aims and significance of this study, as well as the method by which to complete the questionnaires. The participant was completely voluntary and signed written informed consent. Of 5,400 participants recruited from COVID-19 designated hospitals provided 5,271 (97.6%) valid responses. This study was approved by the Ethics Committee of Harbin Sixth Hospital.

### 2.2. Measures

Participants’ data related to social-demographics, dietary behavior, lifestyle, sleep, personality and work-related conflicts were included, providing adequate information of a healthcare worker.

The socio-demographic information included gender, age, marital status, occupation, education, income, length of employment, overtime, frequency of locked down, and COVID-19 vaccines. Healthcare worker—patient conflict was assessed by “Have you ever had any conflict with patients during the COVID-19 pandemic?” Moreover, the participants made a self-report on their BMI changes with the question “Please choose your current BMI group: underweight, normal weight, overweight, or obesity; in addition, according to your weight records, how much has your BMI (kg/m^2^) changed since the COVID-19 outbreak?” Specifically, participants reported their BMI changes independently, and the information of BMI came from weight management APP records. Although the data collected in this survey did not show the specific BMI values before and after the epidemic, these data based on the weight management APP were reliable and acceptable.

The information of dietary behavior and lifestyle among doctors and nurses was collected by interview, including dietary regularity, night eating, skip breakfast, unbalanced nutritional diet, high-fat diet, high-salt diet, high-carbohydrate diet, soft drinks consumption, alcohol consumption, eating out frequency, negative emotional eating, positive emotional eating, overweight or obesity, smoking, and exercise. In the current study, dietary behaviors were assessed with a validated food frequency questionnaire (FFQ), measuring the food consumption of samples in the last 12 months (44). This scale was suitable for Chinese dietary characteristics and could be applied for different scales (45, 46). And then, diet quality was evaluated by the intake of specific nutrients. In the FFQ, “1” was for eating a specific food, while “0” was for not eating such a food. There were 227,099 food items in this study. The “frequency” of food items included four small columns, and only one column was filled in for each food. Fill in the “times/day” column for foods that were consumed more than once a day on average, “times/week” column for foods that were consumed 1∼6 times a week, and “times/month” column for foods that were consumed 1∼3 times a month. Record the average consumption according to the requirements of each food (raw weight, edible part weight), and the unit is g. According to the data from FFQ, there was a corresponding energy and nutrients conversion table. Based on the energy and nutrients conversion table, we calculated the energy and nutrients. A high-fat diet refers to a diet that contains lipids that account for more than 30% of the total energy intake; a high-carbohydrate diet refers to a diet in which carbohydrate accounts for more than 55% of daily energy intake; a high-salt diet refers to a diet in which that the salt intake ≥ 12 g/d (47–49). An “unbalanced nutritional diet” refers to the overall balance of daily diets: on the one hand, a diet contains at least one serving of food per day from each of the five food groups (meat/poultry/fish/egg, dairy/beans, grains, fruits, and vegetables); on the other hand, the balance of a diet is present in the energy-yielding macronutrients in terms of contribution to total energy intake, including macronutrient ratio (carbohydrate: protein: fat) and fatty acid ratio (PUFA: MUFA: SFA). All items were answered by points to measure the level of dietary behaviors. Moreover, Exercise was measured by the question “Do you exercise regularly during the COVID-19 pandemic?”, which was answered by “Never,” “Every day,” “Often 3–5 times/week),” and “Occasionally (1–2 times/month).”

Negative emotional eating and positive emotional eating were measured using the Emotional Eating Scale-Revision (EES-R), which was developed based on the questionnaires designed by Arnow et al. (50). The Chinese version of emotional eating scale contains four dimensions of anger/frustration, anxiety, depression, and positive emotion eating, with a total of 23 items. All items were answered on a 5-point Likert scale to measure the level of binge eating. A higher score indicates a stronger desire to eat. In the present study, Cronbach’s α coefficient for the scale is 0.963, indicating that the EES-R is applicable to this study.

Pittsburgh Sleep Quality Index (PSQI) (51) was used to assess the sleep quality of doctors and nurses. The PSQI assesses seven components of sleep quality, including subjective sleep quality, sleep latency, sleep duration, habitual sleep efficiency, sleep disturbances, use of sleeping medication, and daytime dysfunction. There are 19 questions in this questionnaire, with a total score ranging from 0 to 21. A higher score indicates poorer sleep quality. In the present study, Cronbach’s α coefficient for the questionnaire is 0.948, indicating that the PSQI is applicable to this study. Indeed, this scale was validated for use in Chinese population (52, 53).

Personality traits were measured using the NEO-Personality Inventory Chinese Version, which was developed by Costa and Mccrae (54). This is a well-validated, 25-item self-report instrument for the Chinese population that describes five personality dimensions: neuroticism, extraversion, openness, agreeableness, and conscientiousness. Each item is evaluated on a 5-point Likert scale. Higher scores indicate higher personality trends for the corresponding dimensions. In the present study, Cronbach’s α coefficient for the scale is 0.876, indicating that the NEO-Personality Inventory Chinese Version is applicable to this study.

Burnout symptoms in this study were assessed by a Chinese version of the Maslach Burnout Inventory-General Survey (MBI-GS) (55). There are 15 items in the Chinese version of MBI-GS. The MBI-GS assesses three dimensions of burnout, including emotional exhaustion, depersonalization, personal accomplishment. Each item is evaluated on a 7-point Likert scale. In the present study, all Cronbach’s α coefficients for the three dimensions are higher than 0.900, indicating that the scale is applicable to this study.

Work-Family Conflict Scale (WFCS) (56) was used to evaluate the work-family conflict among Chinese doctors and nurses. There are 18 items rated on a 5-point Likert scale. The WFCS has six dimensions of conflict which consist of time-based work interference with family, time-based family interference with work, strain-based work interference with family, strain-based family interference with work, behavior-based work interference with family, and behavior-based family interference with work. A higher score indicates a higher work-family conflict. In the present study, Cronbach’s α coefficient for the scale is 0.904, indicating that the WFCS is applicable to this study. Indeed, this scale was used in many Chinese researches and showed good reliability and validity (57, 58).

### 2.3. Statistical analysis and machine learning

All analyses were performed in R (version 4.2.1). All tests were two-tailed, with a statistical significance level set at *p* < 0.05.

Descriptive statistics were used to represent the characteristics of doctors and nurses. The human body has a small range of BMI change over several years because of the different measure time, age growth and physiological changes. According to recommended BMI cut-off points for determining underweight, normal weight, overweight, and the previous researches on BMI change (59–61), ± 0.5 kg/m^2^ was set up as normal BMI change range for the stable group in the present study. The participants were categorized into five groups according to the change of BMI: substantial decrease group (BMI change<−2.0), moderate decrease group (BMI change: −2.0 ∼−0.6), stable group (BMI change: −0.5 ∼ 0.5), moderate increase group (BMI change: 0.6∼2.0), substantial increase group (BMI change > 2.0). The characteristics of participants in each group were compared using the Analysis of Variance (ANOVA) test and Chi-square (χ^2^) tests.

This study applied Deep Neural Network to develop a BMI change prediction model among doctors and nurses during the COVID-19 pandemic. H2O package was used for machine learning. DNN is typically used to make a prediction or classification through a series of layers, each of which combines an affine operation and a non-linearity (41). The DNN consists of an input layer, an output layer, and several hidden layers. In the structure, each layer receives the output of the previous layer as its input and passes the output to the next layer. Back-propagation is the dominant algorithm used in training neural networks.

In this study, the output variable was BMI change value, and social-demographics, dietary behavior, lifestyle, sleep, personality traits and work-related conflicts were input variables. Firstly, data were pre-processed with normalization methods. Secondly, we randomly allocated 70% of the sample as the training subset, and 30% of the sample as the test subset. In the training phase of the model development, the training subset was used to generate a learned model prediction. In the validation phase, the model was tested with the test subset which would predict the corresponding outcome. Finally, the performance of BMI change prediction model generated through learning was evaluated by R Squared (*R*^2^), Mean Absolute Error (MAE), Mean Squared Error (MSE), and Root Mean Squared Error (RMSE).

## 3. Results

### 3.1. Sample characteristics

**FIGURE 1:**
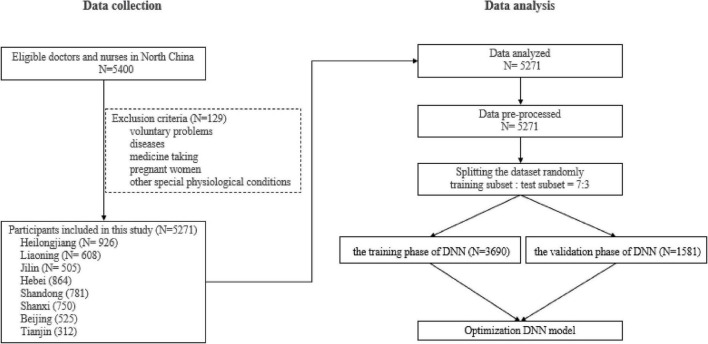
The flowchart of data collection and data analysis.

In this study, a total of 5,271 participants were recruited from 39 COVID-19 designated hospitals in North China (Figure 1).

Table 1 shows the social-demographic information of the samples in the present study. Of these participants, 2,092 (39.7%) were doctors and 3,179 (60.3%) were nurses. 32.5% of participants were males and 67.5% of participants were females. In groups of age < 30, 30–44, 45–59, and ≥ 60 years, there were 753 (14.3%), 2,793 (53.0%), 1,671 (31.7%), and 54 (1.0%) participants, respectively. Of these doctors and nurses, 1,822 (34.6%) had never been locked down due to COVID-19, and 5,107 (96.9%) participants have already been vaccinated against COVID-19. Moreover, in the current study, there were 2,216 individuals keeping a stable BMI, accounting for 42.0%. About the BMI change, the substantial decrease group, moderate decrease group, moderate increase group, and substantial increase group included 217 (4.1%), 1,174 (22.3%),1,244 (23.6%), and 420 (8.0%) participants, respectively. The results of ANOVA test and χ^2^-tests revealed that there were statistically significant differences between five BMI change groups in gender, age, occupation, marital status, education, income, length of employment, overtime, and frequency of locked down (*P* < 0.05).

**TABLE 1:** Social-demographic characteristics of doctors and nurses in North China (*N* = 5,271).

	Total *N* (%)	Substantial decrease group *N* (%)	Moderate decrease group *N* (%)	Stable group *N* (%)	Moderate increase group *N* (%)	Substantial increase group *N* (%)	χ^2^	*P*
Total	5,271 (100.0%)	217 (4.1%)	1,174 (22.3%)	2,216 (42.0%)	1,244 (23.6%)	420 (8.0%)		
Gender							19.597	0.001
Male	1,715 (32.5%)	49 (2.9%)	372 (21.7%)	734 (42.8%)	395 (23.0%)	165 (9.6%)		
Female	3,556 (67.5%)	168 (4.7%)	802 (22.6%)	1,482 (41.7%)	849 (23.8%)	255 (7.2%)		
Age (years)							38.358	0.000
<30	753 (14.3%)	36 (4.8%)	157 (20.8%)	314 (41.7%)	168 (22.3%)	78 (10.4%)		
30–44	2,793 (53.0%)	111 (4.0%)	626 (22.4%)	1,138 (40.7%)	672 (24.1%)	246 (8.8%)		
45–59	1,671 (31.7%)	70 (4.2%)	387 (23.2%)	732 (43.8%)	388 (23.2%)	94 (5.6%)		
≥60	54 (1.0%)	0 (0.0%)	4 (7.4%)	32 (59.3%)	16 (29.6%)	2 (3.7%)		
Occupation							77.804	0.000
Doctor	2,092 (39.7%)	56 (2.7%)	443 (21.2%)	972 (46.5%)	518 (24.7%)	103 (4.9%)		
Nurse	3,179 (60.3%)	161 (5.1%)	731 (23.0%)	1,244 (39.1%)	726 (22.8%)	317 (10.0%)		
Marital status							28.789	0.000
Single	1,148 (21.8%)	53 (4.6%)	203 (17.7%)	474 (41.3%)	300 (26.1%)	118 (10.3%)		
Married	4,123 (78.2%)	164 (4.0%)	971 (23.6%)	1,742 (42.3%)	944 (22.9%)	302 (7.2%)		
Education							1272.468	0.000
Junior college and below	250 (4.7%)	6 (2.4%)	58 (23.2%)	129 (51.6%)	42 (16.8%)	15 (6.0%)		
University	3,129 (59.4%)	179 (5.7%)	913 (29.2%)	1,593 (50.9%)	291 (9.3%)	153 (4.9%)		
Postgraduate and above	1,892 (35.9%)	32 (1.7%)	203 (10.7%)	494 (26.1%)	911 (48.2%)	252 (13.3%)		
Income (yuan/month)							126.859	0.000
<3,000	290 (5.5%)	16 (5.5%)	60 (20.7%)	124 (42.8%)	52 (17.9%)	38 (13.1%)		
3,000–4,999	2,560 (48.6%)	120 (4.7%)	648 (25.3%)	1,112 (43.4%)	468 (18.3%)	212 (8.3%)		
5,000–8,000	1,893 (35.9%)	65 (3.4%)	372 (19.7%)	784 (41.4%)	548 (28.9%)	124 (6.6%)		
>8,000	528 (10.0%)	16 (3.0%)	94 (17.8%)	196 (37.1%)	176 (33.4%)	46 (8.7%)		
Length of employment (years)							104.316	0.000
≤1	184 (3.5%)	12 (6.5%)	44 (23.9%)	88 (47.8%)	36 (19.6%)	4 (2.2%)		
2–4	446 (8.4%)	16 (3.6%)	98 (22.0%)	208 (46.6%)	80 (17.9%)	44 (9.9%)		
5–10	1,168 (22.2%)	28 (2.4%)	240 (20.5%)	524 (44.9%)	252 (21.6%)	124 (10.6%)		
11–15	1,216 (23.1%)	56 (4.6%)	308 (25.3%)	432 (35.5%)	316 (26.0%)	104 (8.6%)		
16–20	493 (9.3%)	33 (6.7%)	116 (23.5%)	196 (39.8%)	100 (20.3%)	48 (9.7%)		
>20	1,764 (33.5%)	72 (4.1%)	368 (20.9%)	768 (43.5%)	460 (26.1%)	96 (5.4%)		
Overtime (hours/week)							89.907	0.000
<5	3,748 (71.1%)	152 (4.1%)	804 (21.5%)	1,672 (44.6%)	840 (22.3%)	280 (7.5%)		
5–10	1,068 (20.3%)	32 (3.0%)	288 (27.0%)	372 (34.8%)	296 (27.7%)	80 (7.5%)		
11–15	190 (3.6%)	12 (6.3%)	38 (20.0%)	72 (37.9%)	52 (27.4%)	16 (8.4%)		
>15	265 (5.0%)	21 (7.9%)	44 (16.6%)	100 (37.8%)	56 (21.1%)	44 (16.6%)		
Frequency of locked down							368.937	0.000
0	1,822 (34.6%)	80 (4.4%)	350 (19.2%)	976 (53.6%)	328 (18.0%)	88 (4.8%)		
1	1,445 (27.4%)	45 (3.1%)	416 (28.8%)	584 (40.4%)	328 (22.7%)	72 (5.0%)		
2	636 (12.0%)	12 (1.9%)	144 (22.6%)	232 (36.5%)	200 (31.5%)	48 (7.5%)		
3	352 (6.7%)	20 (5.7%)	64 (18.2%)	116 (33.0%)	112 (31.8%)	40 (11.3%)		
≥4	1,016 (19.3%)	60 (5.9%)	200 (19.7%)	308 (30.3%)	276 (27.2%)	172 (16.9%)		
COVID-19 vaccines							5.885	0.208
Vaccinated	5,107 (96.9%)	205 (4.0%)	1,134 (22.2%)	2,156 (42.2%)	1,204 (23.6%)	408 (8.0%)		
Unvaccinated	164 (3.1%)	12 (7.3%)	40 (24.4%)	60 (36.6%)	40 (24.4%)	12 (7.3%)		

As shown in Table 2, there were significant differences in the comparison of poor dietary regularity, night eating, skip breakfast, unbalanced nutritional diet, high-fat diet, high-salt diet, high-carbohydrate diet, soft drinks consumption, eating out, negative emotional eating, positive emotional eating, alcohol consumption, exercise, and sleep quality (*P* < 0.05) between the substantial BMI decrease group, moderate BMI decrease group, stable BMI group, moderate BMI increase group, and substantial BMI increase group.

**TABLE 2:** Dietary behaviors, smoking, exercise, and sleep quality among doctors and nurses in North China (*N* = 5,271).

	Total M ± *SD*/*N* (%)	Substantial decrease group M ± *SD*/*N* (%)	Moderate decrease group M ± *SD*/*N* (%)	Stable group M ± *SD*/N (%)	Moderate increase group M ± *SD*/*N* (%)	Substantial increase group M ± *SD*/*N* (%)	*F*/χ^2^	*P*
Poor dietary regularity (points)	1.58 ± 0.712	1.70 ± 0.738	1.55 ± 0.668	1.45 ± 0.649	1.63 ± 0.719	2.13 ± 0.818	91.270	0.000
Night eating (points)	1.74 ± 0.764	1.73 ± 0.696	1.69 ± 0.718	1.68 ± 0.744	1.81 ± 0.784	2.01 ± 0.890	21.030	0.000
Skip breakfast (points)	2.00 ± 1.006	2.07 ± 1.120	1.98 ± 0.975	1.95 ± 1.006	2.05 ± 1.021	2.12 ± 0.964	4.094	0.000
Unbalanced nutritional diet (points)	2.43 ± 0.906	2.61 ± 1.096	2.35 ± 0.858	2.23 ± 0.848	2.65 ± 0.862	2.93 ± 0.999	87.604	0.000
High-fat diet (points)	1.90 ± 0.557	1.85 ± 0.678	1.84 ± 0.570	1.82 ± 0.546	2.00 ± 0.520	2.15 ± 0.494	47.778	0.000
High-salt diet (Points)	1.88 ± 0.567	1.88 ± 0.615	1.81 ± 0.536	1.81 ± 0.574	2.01 ± 0.529	2.05 ± 0.592	39.750	0.000
High-carbohydrate diet (Points)	1.87 ± 0.510	1.78 ± 0.599	1.76 ± 0.502	1.83 ± 0.490	1.98 ± 0.494	2.05 ± 0.524	47.615	0.000
Soft drinks consumption (points)	2.46 ± 0.917	2.46 ± 0.995	2.32 ± 0.873	2.40 ± 0.897	2.59 ± 0.917	2.72 ± 0.992	24.234	0.000
Eating out (points)	2.18 ± 0.939	2.24 ± 1.000	2.10 ± 0.929	2.05 ± 0.929	2.39 ± 0.900	2.42 ± 0.945	36.391	0.000
Negative emotional eating (points)	37.60 ± 14.823	34.00 ± 15.625	36.09 ± 14.077	36.92 ± 14.330	39.12 ± 14.424	42.78 ± 18.193	23.881	0.000
Positive emotional eating (points)	13.91 ± 4.048	14.24 ± 4.411	13.59 ± 3.923	13.38 ± 4.178	14.63 ± 3.569	15.30 ± 4.203	34.791	0.000
Alcohol consumption (Points)	1.44 ± 0.577	1.37 ± 0.522	1.43 ± 0.604	1.40 ± 0.552	1.51 ± 0.600	1.45 ± 0.569	9.038	0.000
Overweight or obesity							8.950	0.062
No	4,534 (86.0%)	187 (4.1%)	1,017 (22.4%)	1,930 (42.6%)	1,039 (22.9%)	361 (8.0%)		
Yes	737 (14.0%)	30 (4.1%)	157 (21.3%)	286 (38.8%)	205 (27.8%)	59 (8.0%)		
Smoking							7.526	0.111
No	4,640 (88.0%)	196 (4.2%)	1,024 (22.1%)	1,936 (41.7%)	1,100 (23.7%)	384 (8.3%)		
Yes	631 (12.0%)	21 (3.3%)	150 (23.8%)	280 (44.4%)	144 (22.8%)	36 (5.7%)		
Exercise							242.850	0.000
Every day	800 (15.2%)	48 (6.0%)	120 (15.0%)	268 (33.5%)	228 (28.5%)	136 (17.0%)		
Often (3–5 times/week)	2,877 (54.6%)	93 (3.2%)	616 (21.4%)	1,248 (43.4%)	712 (24.7%)	208 (7.3%)		
Occasionally (1–2 times/month)	1,172 (22.2%)	60 (5.1%)	320 (27.3%)	468 (39.9%)	268 (22.9%)	56 (4.8%)		
Never	422 (8.0%)	16 (3.8%)	118 (28.0%)	232 (55.0%)	36 (8.5%)	20 (4.7%)		
Poor sleep quality (points)	7.24 ± 4.247	3.41 ± 1.546	3.53 ± 1.513	5.98 ± 3.097	11.50 ± 2.033	13.69 ± 2.500	2570.561	0.000

In addition, Table 3 indicates that there were significant differences in comparison of personality traits (neuroticism, extraversion, openness, agreeableness, conscientiousness), emotional exhaustion, depersonalization, personal accomplishment, healthcare worker—patient conflict, and work-family conflict (*P* < 0.05) between the substantial BMI decrease group, moderate BMI decrease group, stable BMI group, moderate BMI increase group, and substantial BMI increase group.

**TABLE 3:** Personality traits, burnout, and work-related conflicts among doctors and nurses in North China (*N* = 5,271).

	Total M ± *SD*/N (%)	Substantial decrease group M ± *SD*/*N* (%)	Moderate decrease group M ± *SD*/*N* (%)	Stable group M ± *SD*/*N* (%)	Moderate increase group M ± *SD*/*N* (%)	Substantial increase group M ± *SD*/*N* (%)	*F*/χ^2^	*P*
Neuroticism (points)	55.39 ± 12.791	53.71 ± 13.874	54.66 ± 12.933	56.60 ± 13.496	54.81 ± 11.362	53.65 ± 11.388	9.446	0.000
Extraversion (points)	45.72 ± 9.168	48.35 ± 9.274	45.12 ± 9.334	45.71 ± 9.711	45.80 ± 8.168	45.88 ± 8.227	5.816	0.000
Openness (Points)	55.30 ± 10.599	56.09 ± 11.098	55.13 ± 10.569	56.03 ± 11.163	54.54 ± 9.582	53.79 ± 9.915	6.732	0.000
Agreeableness (Points)	47.75 ± 12.336	50.34 ± 13.765	47.20 ± 12.187	48.47 ± 12.814	46.81 ± 11.665	46.99 ± 10.890	7.083	0.000
Conscientiousness (points)	38.82 ± 10.896	42.09 ± 11.720	38.81 ± 10.643	38.72 ± 11.816	38.04 ± 9.461	40.10 ± 9.625	8.036	0.000
Emotional exhaustion (points)	15.71 ± 7.243	18.32 ± 8.804	15.56 ± 6.886	13.97 ± 6.62993	16.69 ± 6.620	21.02 ± 8.609	109.465	0.000
Depersonalization (points)	9.40 ± 5.693	10.65 ± 6.618	9.09 ± 4.937	8.46 ± 5.255	9.87 ± 5.615	13.18 ± 7.524	70.633	0.000
Personal accomplishment (points)	31.69 ± 9.652	33.32 ± 8.102	31.20 ± 9.948	31.45 ± 10.134	32.23 ± 8.907	31.86 ± 8.913	3.640	0.006
Healthcare worker—patient conflict							66.955	0.000
Never occurred	4,130 (78.4%)	152 (3.7%)	926 (22.4%)	1,836 (44.5%)	924 (22.3%)	292 (7.1%)		
Occurred	1,141 (21.6%)	65 (5.7%)	248 (21.7%)	380 (33.4%)	320 (28.0%)	128 (11.2%)		
Work-family conflict (points)	49.94 ± 10.768	53.61 ± 8.951	49.08 ± 10.260	48.13 ± 10.889	51.53 ± 9.938	55.25 ± 11.854	58.532	0.000

### 3.2. BMI change prediction model in Chinese doctors and nurses

**FIGURE 2:**
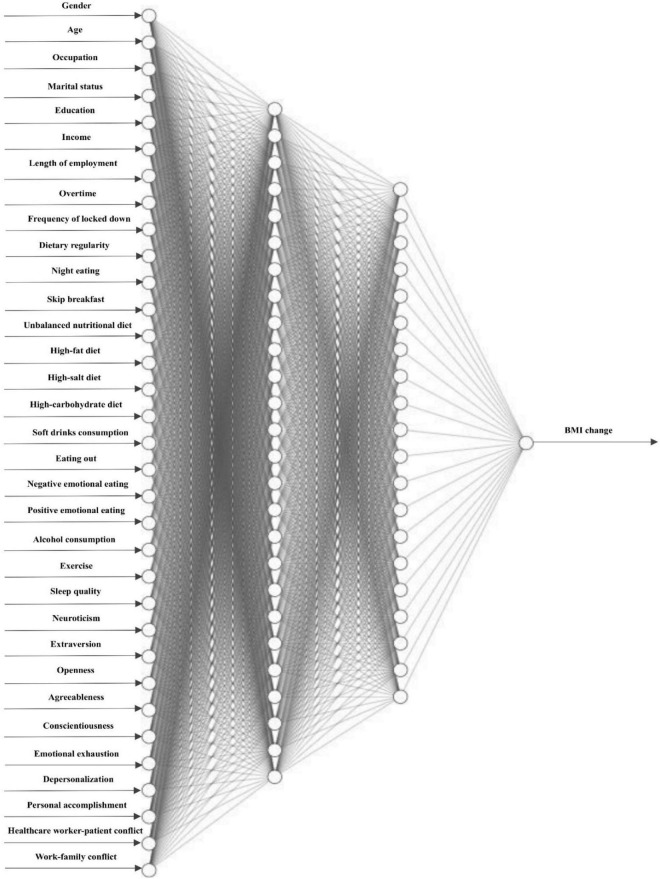
Deep neural network for BMI change prediction.

Based on the results of ANOVA test and χ^2^-tests, this study further developed a Deep Neural Network (DNN) model with a 33-26-20-1 network framework for BMI change prediction, which is presented in Figure 2. For the DNN model, values of *R*^2^, MAE, MSE, and RMSE were 0.940, 0.027, 0.002, 0.038, respectively.

**FIGURE 3:**
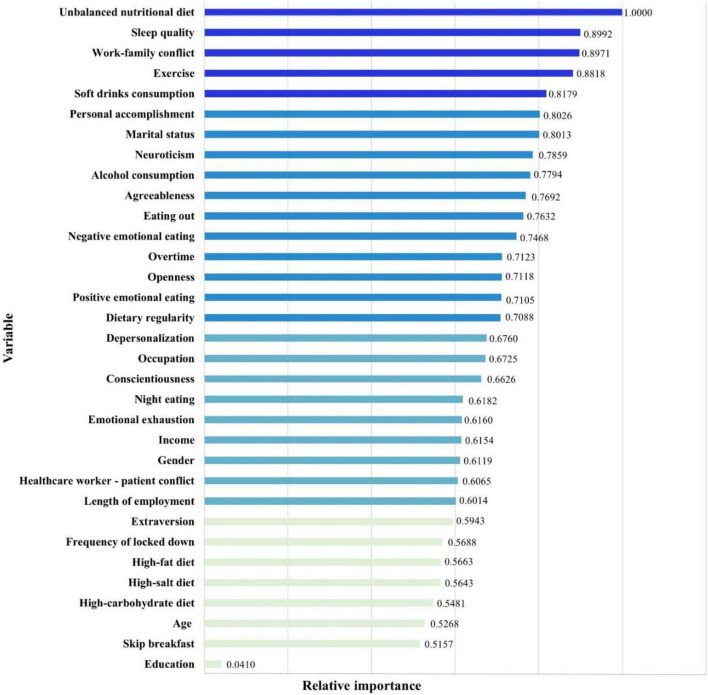
Relative importance of each predictor in the BMI change prediction model.

In addition, as Figure 3 showed, the results of H2O deep learning also revealed the relative importance of each predictor. Among doctors and nurses in China, the top five predictors in the BMI change prediction model were unbalanced nutritional diet, poor sleep quality, work-family conflict, lack of exercise, and soft drinks consumption.

## 4. Discussion

The COVID-19 pandemic has become a major public health concern over the past 3 years, put extraordinary pressure on the front-line healthcare workers, and led to adverse effects on their mental and physical health. This study recruited doctors and nurses from COVID-19 designated hospitals in North China as samples. Of 5,271 participants, only 2,216 individuals kept a stable BMI, accounting for 42.0%. There were statistically significant differences between five BMI change groups (substantial decrease, moderate decrease, stable, moderate increase, substantial increase) in socio-demographic, personality traits, dietary behavior, emotional eating, lifestyle, sleep quality, burnout, and work-related conflicts. Furthermore, in an increasingly data-driven world, machine learning is expected to be a key tool for converting big data into tangible benefits, particularly in healthcare settings. A deep neural network (DNN) is able to identify complex patterns in multi-dimensional data and use these uncovered patterns to classify new unseen cases or make data-driven predictions. Hence, this study developed a BMI change prediction model using a machine learning method, and identified key variables within the data contributing to BMI change risk among doctors and nurses during the COVID-19 pandemic. Results showed the developed ensemble model for BMI change with a 33-26-20-1 network framework. The DNN model achieved high prediction efficacy, and values of *R*^2^, MAE, MSE, and RMSE for the model were 0.940, 0.027, 0.002, 0.038, respectively. Among doctors and nurses in China, the top five predictors in the BMI change prediction model were unbalanced nutritional diet, poor sleep quality, work-family conflict, lack of exercise, and soft drinks consumption.

Results of this study indicated that dietary behaviors were the most important predictor of the prevalence of BMI change among doctors and nurses in China. In particular, unbalanced nutritional diet was the strongest predictor in the BMI change prediction model. Front-line doctors and nurses had additional challenges when they adjusted to an entirely new working environment in this stressful situation during the COVID-19 pandemic. The current dietary situation of Chinese doctors and nurses was pessimistic. Due to unstable working hours, work assignments and work settings, they exhibited increased unhealthy dietary behaviors such as skipping breakfast, night eating, ready-prepared meal consumption, eating out, emotional eating, soft drinks consumption, poor dietary regularity, and unbalanced nutritional diet, etc. Although changes of body weight and BMI are generally considered to be caused by the imbalances between calorie intake and expenditure, a review suggested that differences of impact on weight management between low-fat diets, low-carbohydrate diets, and Mediterranean approaches were marginal (62). A more scientific and balanced nutritional diet is the key to weight management. Scholars have conducted many studies, and found that various components of energy balance were dynamically interrelated and weight change was resisted by counterbalancing physiological processes (63). Unbalanced nutritional diet was characterized by low cereals, vegetables, fruits, micronutrient density, and excessive of highly processed, sweet, high-fat foods, red meat and derivatives, as well as extreme diets (64, 65). For individuals with increased BMI, unbalanced nutritional diet shows more food with high calories and overeating; while nutrient and caloric intakes were significantly reduced for individuals with decreased BMI. Moreover, as results of the current study showed, soft drinks consumption was the fifth most important predictor of BMI change during the COVID-19 pandemic. The consumption of soft drinks, including carbonated drinks, juice drinks, sports and energy drinks, has increased dramatically over the past several years. Researches suggested that soft drinks were high in sugar content and acidity, which had little nutritional benefit and lacked micro-nutrients, vitamins and minerals, only providing energy (66). Soft drinks consumption contributes to dental caries, tooth erosion, and general health such as overweight and obesity. Soft drinks not only have a negative impact on body weight, but also increase the risk of chronic diseases. Therefore, restricting the intake of soft drinks has become one of the global public health policies to reduce the potential harmful effects of soft drinks on general health in populations.

Poor sleep quality was the second most crucial predictor of BMI change among doctors and nurses in China. Due to the increased work pressure during the COVID-19 pandemic, sleep problems of healthcare workers have been increasingly serious. Especially in China, the COVID-19 epidemic has posed new severe challenges to the national people. Under the government-led prevention and control of the epidemic, designated hospitals and medical staff are genuinely ready to treat patients suffering from the virus. For Chinese doctors and nurses, 24 on call during the COVID-19 epidemic is an inevitable choice for the sacred mission of medicine, a responsible working attitude, and an unswerving belief in patients’ safety. The association between poor sleep quality and risk of weight and BMI change is well established for all age groups. A study focused on sleep and weight-height development has showed that insomnia was the most prevalent disorder, and shorter sleep duration was associated with the development of overweight and obesity (67). Several clinical studies provided underlying mechanisms of the relationship between sleep duration and weight change: food intake, appetite-regulating hormones, emotion regulation, and energy expenditure (68). Given the negative consequences of poor sleep quality for weight management, Chinese doctors and nurses should develop healthy sleep habits in their daily work-life guidelines as an adjuvant in preventing and managing weight and BMI changes.

Particularly, although previous studies have confirmed the role of work-family conflict, few studies have incorporated it into physical symptoms prediction models (69, 70). The current study suggested that work-family conflict greatly impacted the BMI change of Chinese doctors and nurses, ranking third in the DNN prediction model. As discussed above, the COVID-19 pandemic has placed increased stress on front-line healthcare workers, leading them to greater risk for worsening mental and physical health. The heavy workload made it difficult for healthcare workers to take care of the families, including being responsible for children and parents, and household tasks. An epidemiological study provided evidence that individuals with imbalanced work-family relationships had an increased risk of unhealthy eating styles and maladaptive physical outcomes (71). Work-family conflict is a potential variable in explaining unhealthy eating, weight change, as well as a tendency to emotional eating because of stress, anxiety, fear, depression, etc. According to the current study, work-family conflict led to an increased risk of BMI change among doctors and nurses during the COVID-19 pandemic. Therefore, Chinese policymakers and hospital administrators should implement effective strategies to support the balance of healthcare workers’ work-family life.

Meanwhile, exercise has a lot of benefits for body weight management, which is consistent with the results of this study. During the COVID-19 pandemic, this study indicated that lack of exercise was the fourth most important predictor of BMI change among doctors and nurses in China. As a clinical nutrition study showed, exercise resulted in energy balance, healthier body composition, and body weight management (72). Compared with dietary behaviors, exercise training has effects on preferential loss of body fat and maintenance of fat-free mass. Additionally, the impact of COVID-19 on healthcare workers is not only high work pressure, but also a variety of emotional and psychological problems, such as nervousness, fear, anxiety, depression, emotional exhaustion, depersonalization, and low personal accomplishment. Exercise can improve emotional function and mental health *via* the regulation of dopamine (73), and further reduce unhealthy dietary behaviors including emotional eating. All in all, exercise benefits human mental and physical health, as well as a modest degree of average weight loss. Lack of exercise may lead to overweight or underweight, and insufficient muscle strength. Note that individual responses are highly variable in the impact of exercise on weight management. Due to a lack of free time and unstable work hours, front-line healthcare workers relatively lacked physical activity during the COVID-19 pandemic. Front-line doctors and nurses should raise awareness about the benefits of exercise, and exercise as often as possible to improve weight management.

Overall, the current study sheds more light on the prediction of BMI change among Chinese doctors and nurses during the COVID-19 pandemic using a large sample size of 5,271 participants from COVID-19 designated hospitals. Meanwhile, personality traits, dietary behaviors, lifestyles, sleep quality, burnout, and work-related conditions have contributed to the BMI change prediction. The policymakers, hospital administrators and healthcare workers should take integrated treatment measures in the management of weight and BMI, including healthy dietary behaviors, regular exercise, sleep quality improvement, and work-related conditions promotion.

### 4.1. Limitations

This article has several notable limitations. First, the study samples were recruited from COVID-19 designated hospitals in North China, leading to a potential sampling bias. And this study did not count the number of potential participants who were approached but did not accept to participate in this survey. Future studies should examine the generalization of these findings in other healthcare workers populations. Second, the application of self-reporting measurements could represent a limitation of the study because of the possible underestimated status of social-demographics, dietary behavior, lifestyle, sleep, personality and work-related conflicts among Chinese doctors and nurses during the COVID-19 pandemic. In fact, participants reported their BMI changes independently, and the information of BMI came from weight management APP records. The data collected in this survey did not show the specific BMI values before and after the epidemic, which might lead to information bias. Third, given a limited number of input variables in the BMI change prediction model, information of policy and environment should be included in future studies.

## 5. Conclusion

The COVID-19 pandemic has become a major public health concern over the past 3 years, leading to adverse effects on front-line doctors and nurses. BMI change was highly prevalent (58.0%) among doctors and nurses in North China. Machine learning models can provide an automated identification mechanism for the prediction of BMI change. In the Deep neural network for BMI change prediction, the top five predictors were unbalanced nutritional diet, poor sleep quality, work-family conflict, lack of exercise, and soft drinks consumption. The policymakers, hospital administrators and healthcare workers should take integrated treatment measures in the management of weight and BMI, to provide the scientific basis for mental and physical health improvement among doctors and nurses during the COVID-19 pandemic.

## Data availability statement

The raw data supporting the conclusions of this article will be made available by the authors, without undue reservation.

## Ethics statement

The studies involving human participants were reviewed and approved by the Ethics Committee of Harbin Sixth Hospital. The patients/participants provided their written informed consent to participate in this study.

## Author contributions

AL designed the study. QW and HC drafted the protocol, analyzed the data, and wrote the manuscript. PQ, HF, DL, SL, and JL participated in the data collection and analyzed and discussed the results. All authors have reviewed the final manuscript and approved it for publication.
